# Modeling parasitoid development: climate change impacts on *Telenomus remus* (Nixon) and *Trichogramma foersteri* (Takahashi) in southern Brazil

**DOI:** 10.1002/ps.8888

**Published:** 2025-05-08

**Authors:** Fábio Sampaio, Cesar A. Marchioro, Luís A. Foerster

**Affiliations:** ^1^ Graduate Program in Plant Production ‐ Agronomy, Department of Plant Protection Federal University of Paraná Paraná Brazil; ^2^ Graduate Program in Natural and Agricultural Ecosystems, Department of Agriculture, Biodiversity, and Forests Federal University of Santa Catarina (UFSC) Curitibanos Brazil

**Keywords:** biological control, egg parasitoids, voltinism, development rate, global warming

## Abstract

**BACKGROUND:**

The egg parasitoids *Telenomus remus* (Nixon) and *Trichogramma foersteri* (Takahashi) were recently collected in southern Brazil, expanding their potential use in biological control. Understanding how these species respond to temperature is essential to the effective implementation of biological control programs, especially in the context of global warming. In this study, phenological models were employed to assess the effects of temperature and climate change on their development.

**RESULTS:**

Temperature had a significant impact on the development of *Te. remus*, with development times ranging from 52.7 days at 15 °C to 8.1 days at 35 °C. Parasitism peaked at 35 °C (124.15 eggs) and lowest at 15 °C (38.5 eggs). Emergence rates declined under extreme temperatures, especially at 15 °C. The Brière‐2 and Shi models were identified as the most appropriate for *Te. remus* and *T. foersteri*, respectively. Under the SSP2‐4.5/2080 scenario, an increase in the number of generations was projected. In contrast, in the SSP5‐8.5 scenario, higher temperatures may exceed the thermal thresholds of these species, potentially reducing voltinism in warmer regions while promoting it in colder areas.

**CONCLUSION:**

*Telenomus remus* and *T. foersteri* exhibit broad thermal tolerance; however, extreme temperatures, including those predicted under climate change scenarios, can restrict their development. This study offers valuable insights for laboratory rearing programs, mass production, and field release programs while enhancing the understanding of thermal interactions in Hymenopteran parasitoids. © 2025 The Author(s). *Pest Management Science* published by John Wiley & Sons Ltd on behalf of Society of Chemical Industry.

## Graphical abstract

Phenological models revealed temperature‐driven development variability in *Telenomus remus* and *Trichogramma foersteri*, highlighting potential impacts of climate change on their biological control efficacy in warmer and colder regions.

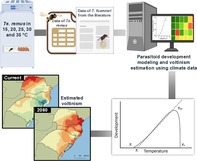

## 1 INTRODUCTION

The use of egg parasitoids for the biological control of insect pests is becoming increasingly common in modern agriculture.^1^, ^2^, ^3^ In recent years, biological control has gained particular relevance in the management of *Spodoptera spp*., especially in *Bt* crops expressing *Bacillus thuringiensis* (Berliner) toxins, where insecticide applications are less frequent.^4^ Parasitoid wasps of the order Hymenoptera rank among the most important natural enemies of insect pests.^5^ Several species of micro‐wasps from the genera *Trichogramma* (Westwood) and *Telenomus* (Haliday) are employed to control a broad range of pests in various regions worldwide.^2^, ^3^, ^6^, ^7^

The genus *Trichogramma* is notable for its widespread use, primarily because these parasitoids can be easily mass‐reared in factitious hosts, facilitating large‐scale production for field release.^2^ Conversely, some *Telenomus* species still lack well‐established mass‐rearing techniques.^6^ Despite this, numerous reports document the natural occurrence of *Telenomus* species in crop fields, where they contribute to pest control in these ecosystems.^6^, ^8^, ^9^

The species *Trichogramma foersteri* (Takahashi) was recently identified in southern Brazil.^10^ Several studies have highlighted its high parasitism potential against various agricultural pests.^10^, ^11^, ^12^, ^13^ In contrast to *T. foersteri*, *Telenomus remus* (Nixon) is a well‐known and extensively studied species worldwide.^6^, ^8^ More than 35 years after the introduction of *Te. remus* in Brazil, the species was recently collected from *Spodoptera cosmioides* (Walker) eggs in the same region where *T. foersteri* was found.^4^ This discovery supports the hypothesis that the parasitoid can parasitize multiple species and sustain its population in the field.

The field capture of *T. foersteri* and *T. remus* opens new possibilities for the use of these parasitoids in Brazil. These findings provide valuable opportunities for further bioecological research on both species, which are crucial for biological control programs.^14^ Notably, the collection area for these species experiences well‐defined seasons, characterized by warm summers and harsh winters.^15^, ^16^ Such climatic conditions may challenge the parasitoid's survival, motivating an investigation into the influence of temperature on their development, as temperature is considered the primary abiotic factor affecting insect growth.^17^, ^18^ While a previous study evaluated the effects of temperature on the biology of *T. foersteri*,^19^ no information is currently available regarding the lineage of *Te. remus* collected in southern Brazil.

Phenological models are increasingly used to understand how temperature affects insect development,^18^, ^20^, ^21^ although research on egg parasitoids remains scarce. Fitting multiple functions to developmental data across different temperatures is crucial for understanding parasitoid biology,^20^ particularly in the case of *T. foersteri*, for which fundamental biological studies are still lacking. This knowledge has practical applications for developing laboratory rearing protocols aimed at mass production for commercial purposes, as well as for assessing the potential effects of climate change on parasitoids.^18^, ^22^, ^23^

Climate change poses a significant challenge for parasitoids, as it is expected to substantially impact trophic interactions depending on species‐specific biology and ecology.^24^, ^25^ In this context, determining the thermal thresholds that parasitoids can tolerate is essential for understanding how global warming may influence their survival and development. This knowledge is critical for guiding the management of these species in the coming years, considering that climate change could alter their capacity to persist in current habitats.^26^, ^27^ Despite its importance, there is a notable lack of studies evaluating the impacts of climate change on parasitoids, as most research focuses on insect pests. While projections generally indicate an increase in pest outbreaks and new invasions, few models account for the impact of climate change on natural enemies.^24^ In this context, climate change could not only reshape existing biological control programs but also create new opportunities for using parasitoids, depending on the species and region involved. Therefore, this study aimed to evaluate the effect of temperature on the development of *Te. remus*. Various phenological models were fitted to the data generated for *Te. remus* and to data from the literature for *T. foersteri*,^19^ with the most appropriate models for each parasitoid selected. These models were subsequently used to estimate the number of generations and the days when temperatures exceed the thermal thresholds tolerated by each species, both under current conditions and across different climate change scenarios.

## 2 MATERIALS AND METHODS

### 2.1 Obtaining and rearing *Te. Remus* and its host

Specimens of *Te. remus* were collected from parasitized eggs of *Spodoptera cosmioides* (Walker) in soybean crops (*Glycine max* L. Merril) during the 2018/19 growing season in São José dos Pinhais (25°36′49.0”S, 49°08′01”W), Paraná, Brazil.^4^ The fall armyworm (*Spodoptera frugiperda* (J. E. Smith)), the natural host of *Te. remus*, was collected from a maize crop during the same growing season at the Canguiri Experimental Farm in Pinhais (25°24′01″S, 49°07′01″W), Paraná, Brazil. The collected insects were maintained in the laboratory under controlled conditions of temperature, humidity, and photoperiod (25 ± 1 °C, 70 ± 10% RH, and a 14:10 light/dark cycle). Rearing of *Te. remus* followed the methodology described in the literature with adaptations.^28^ The parasitoid was reared on *S. frugiperda* eggs, which were kept in the laboratory according to established protocols^28^ and fed with an artificial diet.^29^

### 2.2 Effect of temperature on the development and survival of *Te. remus*

The experiments were conducted using a completely randomized design, with five treatments (constant temperatures) and 20 replicates per treatment. The effect of temperature on the development time and survival of *Te. remus* was evaluated in climate‐controlled chambers set to constant temperatures of 15, 20, 25, 30, and 35 ± 1 °C, 70 ± 10% RH, and a 14:10 light/dark cycle. At each temperature, 20 glass tubes (7.5 cm in length and 1.0 cm in diameter) were used, each containing egg masses of approximately 200 *S. frugiperda* eggs (less than 24 h old), fixed onto cardstock rectangles (6 × 1.5 cm). A single mated female *Te. remus*, less than 24 h old and with no prior exposure to eggs, was placed in each tube to parasitize the egg mass for 24 h. Drops of honey were provided as food inside the tubes, which were sealed with cotton. After the parasitism period, the females were removed. Emerging *S. frugiperda* larvae from non‐parasitized eggs were counted and removed twice daily to prevent cannibalism. Following parasitoid emergence, the egg masses were examined under a stereoscopic microscope to determine the number of parasitized eggs, the emergence rate, and the sex of the emerged parasitoids.

At each temperature, the following parameters were evaluated: development time (from egg to adult), number of parasitized eggs (total number of darkened eggs), emergence rate (number of eggs with exit holes/total number of darkened eggs × 100), sex ratio (number of females/total number of adults), adult longevity, and survival of *Te. remus* adults. Adult longevity was assessed using 20 newly emerged pairs at 25 °C, each pair individually placed in glass tubes (7.5 cm in height × 1.0 cm in diameter) containing pure honey as food, without exposure to eggs. The tubes were maintained at the specified temperatures, with daily observations to record the death date of both males and females.

### 2.3 Statistical analysis

Parameters related to the development of *Te. remus* at different temperatures were analyzed in the R computational environment,^30^ using generalized linear models with the *multcomp* package.^31^ Development time was modeled with a Gamma distribution (inverse link function). A quasi‐Poisson distribution (log link function) was applied for the number of parasitized eggs and adult longevity, while logistic regressions with a quasi‐binomial distribution (logit link function) were used for the emergence rate and sex ratio. The chi‐square test of independence was used to assess the effects of temperature on the evaluated parameters. When significant differences were detected between treatments, post‐hoc comparisons were conducted using Tukey's test,^32^ at a 5% significance level with the *emmeans* package.^33^ Survival curves for each temperature were generated from the longevity data of females and males and compared using the non‐parametric Kaplan–Meier method^34^ with the *survival* package.^35^

### 2.4 Selection of mathematical models

The development time data for *Te. remus* and *T. foersteri* at each temperature were used to estimate the thermal development thresholds of both species. Data on the development of *T. foersteri* at the constant temperatures of 15, 20, 25, 28 and 30 °C were obtained from the literature.^19^ Twelve commonly used models from the literature were evaluated, including one linear and 11 non‐linear models, which were fitted to the observed development rate (1/development time) across the tested temperatures for each parasitoid. The models and their estimated parameters are presented in Table 1.

**Table 1:** Mathematical models employed to describe the temperature‐dependent development rate of *Telenomus remus* and *Trichogramma foersteri*

Model	Function^†^	Reference
Linear	$D\left(T\right)=a+b*T$	Campbell *et al*. (1974)^36^
Beta‐16	$D\left(T\right)=c_{m}\;{\left[{\left(\frac{T_{H}-T}{T_{H}-T_{\mathrm{opt}}}\right)\left(\frac{T-T_{L}}{T_{\mathrm{opt}}-T_{L}}\right)}^{\left(T_{\mathrm{opt}}-T_{b}\right)/\left(T_{H}-T_{\mathrm{opt}}\right)}\right]}^{\delta }$	Shi *et al*. (2016)^60^
β type	$D\left(T\right)=\rho ∙\left(a\frac{T}{10}\right)∙{\left(\frac{T}{10}\right)}^{\beta }$	Damos *et al*. (2008)^61^
Brière‐1	$D\left(T\right)=\mathrm{aT}\left(T-T_{L}\right){\left(T_{H}-T\right)}^{1/2}$	Brière *et al*. (1999)^37^
Brière‐2	$D\left(T\right)=\mathrm{aT}\left(T-T_{L}\right){\left(T_{H}-T\right)}^{1/m}$	Brière *et al*. (1999)^37^
Kontodimas	$D\left(T\right)=\alpha {\left(T-T_{L}\right)}^{2}\left(T_{H}-T\right)$	Kontodimas (2004)^39^
Lactin‐1	$D\left(T\right)=e^{\left(\mathrm{\rho T}\right)}-e^{\left(\rho T_{H}-\frac{T_{H}-T}{∆T}\right)}$	Lactin *et al*. (1995)^62^
Lactin‐2	$D\left(T\right)=e^{\left(\mathrm{\rho T}\right)}-e^{\left(\rho T_{H}-\frac{T_{H}-T}{∆T}\right)}+\lambda$	Lactin *et al*. (1995)^62^
Logan‐6	$D\left(T\right)=\varphi \left[e^{\left(\mathrm{\rho T}\right)}-e^{(\rho T_{H}-\frac{\left(T_{H-}T\right)}{∆T}}\right]$	Logan *et al*. (1976)^63^
Performance‐2	$D\left(T\right)=c\left(T-T_{L}\;\right)\left(1-e\left(K\left(T-T_{H}\right)\right)\right)$	Shi *et al*. (2011)^64^
Shi	$D\left(T\right)=c\left(1-e\left[-K1\left(T-T_{L}\right)\right]\right)\left(1-e\left[K2\left(T-T_{H}\right)\right]\right)$	Shi *et al*. (2011)^64^
Taylor	$D\left(T\right)=\mathrm{Rm}\;e\left\{-0,5{\left[\frac{\left(T-T_{\mathrm{opt}}\right)}{T_{L}}\right]}^{2}\right\}$	Taylor (1981)^65^

^†^ *T*_*L*_. *T*_*H*_, *T*_*opt*_ are, respectively, the lower temperature threshold, upper temperature threshold and the optimum temperature for development (°C), *K* is the thermal constant. The remaining parameters are fitted coefficients.

The linear model estimates the lower thermal threshold (T_L_) and the thermal constant (*K*) using the x‐axis intersection method (T_L_ = *−a/b*) and the reciprocal slope calculation (*K* = 1/*b*), respectively.^36^ Non‐linear models are more complex due to their larger number of parameters and, in addition to describing the relationship between temperature and development rate, some can also explain the physiological and biochemical mechanisms underlying insect responses to temperature.^37^ Therefore, besides being evaluated using statistical criteria to assess the quality of fit to the observed data, the models were also assessed for their ability to accurately estimate biologically meaningful parameters.^37^, ^38^, ^39^, ^40^ All model parameters were estimated using the *devRate* package^41^ in the R computational environment,^30^ employing the Levenberg–Marquardt algorithm.

### 2.5 Evaluation of mathematical models

#### 2.5.1 Statistical criteria

The quality of the models was evaluated using regression error (S), Akaike Information Criterion (AIC),^42^ and Bayesian Information Criterion (BIC).^43^ AIC is widely used to assess the relative quality of models by balancing complexity (number of parameters) with goodness of fit. BIC, another statistical tool used for model selection, is partially based on the likelihood function and closely related to AIC. Models with lower S, AIC, and BIC values were considered more suitable.^44^, ^45^ Additionally, ΔAIC and ΔBIC (differences between the lowest AIC and BIC values for each model) were calculated, with values ≤2 indicating comparable model performance.^46^ These criteria were calculated using the *devRate* package^41^ in R.^30^

#### 2.5.2 Biologically meaningful parameters

The lower (T_L_) and upper (T_H_) thermal thresholds, optimal temperature (T_opt_), and thermal constant (K) were considered biologically meaningful parameters.^37^, ^39^, ^40^ Observed laboratory data for both species were compared with values predicted by each model. For *Te. remus*, thermal thresholds outside the ranges between 6.0 and 15.0 °C for T_L_, 32.0 and 34.0 °C for T_opt_, and 34.0 and 36.0 °C for T_H_ were regarded as unrealistic. For *T. foersteri*, thresholds outside 8.0 and 15.0 °C for T_L_, 30.0 and 32.0 °C for T_opt_, and 32.0 and 33.0 °C for T_H_: were considered unrealistic. Preference was given to models that estimated thermal thresholds within these expected ranges.

### 2.6 Annual generation estimates

#### 2.6.1 Study area

The number of annual generations of *Te. remus* and *T. foersteri* were estimated under current climatic conditions and future projections for southern Brazil. This region, covering approximately 576 774 km^2^, encompasses humid subtropical and oceanic climates, according to the Köppen classification.^15^ Mountainous areas in Santa Catarina and Rio Grande do Sul are characterized by colder climates, mild summers, and harsh winters, with frequent frosts and occasional snowfall.^16^ Conversely, warmer regions, such as northwestern Paraná and parts of Rio Grande do Sul, experience hot summers and mild winters.

#### 2.6.2 Climate data

Daily climatic data representative of current conditions for simulate parasitoid voltinism, were obtained from the literature (https://utexas.box.com/Xavier-etal-IJOC-DATA),^47^ covering 908 grids of 0.25° each across the entire study area. These data include 20 years (1994–2013) of minimum and maximum daily air temperatures, interpolated from meteorological stations.^47^ For each grid and day of the year, a 20‐year average of the climatic data was calculated prior to its use in voltinism estimations.

To assess future warming in the study area, data were obtained from the Sixth Assessment Report of the IPCC on climate change (2021)^48^ and sourced from the WorldClim version 2.1 database (https://worldclim.org).^49^ These data included current climate conditions (1970–2000)^50^ as well as projections for 2040 and 2080 based on SSP2‐4.5 and SSP5‐8.5 climate change scenarios. SSP2‐4.5 predicts an intermediate emissions scenario, with projected global warming of 2.0 °C for 2041–2060 and 2.7 °C for 2081–2100. SSP5‐8.5 is a high emissions scenario, with projected global warming of 2.4 °C for 2041–2060 and 4.4 °C for 2081–2100.^48^

Projections were based on four GCMs: BCC ‐ Beijing Climate Center; MIROC6 ‐ Model for Interdisciplinary Research on Climate; CMCC‐ESM2 ‐ Euro‐Mediterranean Center on Climate Change ‐ Earth System Model; and CNRM‐CM6‐1 ‐ Centre National de Recherches Météorologiques ‐ Climate Model 6.1. To reduce uncertainties, the average of the four GCMs was calculated, generating consensus maps for each year and climate scenario. The magnitude of warming in the area was estimated by calculating the differences between future and current mean annual temperatures (in °C) using the R statistical environment.^30^ These differences, specific to each grid cell and climate scenario, were then added to the minimum and maximum daily air temperatures^47^ to account for regional variations in global warming.^51^

#### 2.6.3 Voltinism of *Te. remus* and *T. foersteri*

The best models selected in the previous step were used to calculate voltinism. In this approach, the daily development rate was calculated as the average of the values obtained from the minimum and maximum air temperatures. These daily development rates were then accumulated over consecutive days, with a complete generation considered when the total development reached a value of 1. The voltinism of *Te. remus* and *T. foersteri* was estimated using current and future climatic data, with the egg‐to‐adult lifecycle considered as a complete generation. All calculations were performed in the R computational environment.^30^

#### 2.6.4 Days exceeding thermal thresholds of *Te. remus* and *T. foersteri*

To simulate the impact of current and future climatic conditions on parasitoid development, the thermal threshold estimates (lower threshold, T_L_, and upper threshold, T_H_) from the selected models were applied. The number of days per year with daily minimum temperatures below T_L_ and daily maximum temperatures above T_H_ was analyzed using daily climatic data for the study area. This analysis provides insights into potential climatic constraints and favorable periods for the development of *Te. remus* and *T. foersteri*, offering projections of how shifts in temperature regimes might influence their biological cycles.

## 3 RESULTS

### 3.1 Effect of temperature on the development and survival of *Te. remus*

Temperature significantly influenced the development time of *Te. remus* (χ^2^ = 110 203; df = 4; *P* < 0.001), with an inverse relationship between temperature and development duration. The development time ranged from 52.70 ± 0.41 days at 15 °C to 8.10 ± 0.03 days at 35 °C (Table 2).

**Table 2:** Development (mean ± standard error) of *Telenomus remus* at different constant temperature regimes parasitizing *Spodoptera frugiperda* eggs

Temperature (°C)	Egg‐adult duration (days)^†^	Eggs parasitized^†^	Emergence rate (%)^†^	Sexual ratio (%)^†^
15	52.70 ± 0.41 a	38.50 ± 2.91 e	15.29 ± 2.95 b	0.94 ± 0.03 a
20	21.09 ± 0.11 b	106.45 ± 3.51 d	93.73 ± 0.90 a	0.87 ± 0.01 ab
25	12.49 ± 0.03 c	110.95 ± 6.14 c	95.15 ± 0.95 a	0.83 ± 0.01 b
30	8.26 ± 0.02 d	120.60 ± 7.35 b	96.28 ± 1.43 a	0.82 ± 0.02 b
35	8.10 ± 0.03 e	124.15 ± 5.02 a	78.16 ± 2.05 a	0.86 ± 0.01 b

^†^ Means followed by the same letters within the columns are not statistically different according to Tukey test at a significance level of 5%.

The number of eggs parasitized by *Te. remus* varied significantly with temperature (χ^2^ = 227.46; df = 4; *P* < 0.001). Parasitism peaked at 35 °C (124.15 eggs) and was lowest at 15 °C (38.50 eggs). Similarly, temperature significantly influenced the emergence rate of *Te. remus* (χ^2^ = 671.64; df = 4; *P* < 0.001), with lower rates observed at extreme temperatures, particularly at 15 °C, where only 15.29% of parasitized eggs emerged. However, no significant differences in emergence rates were detected among the other temperatures tested (Table 2). Additionally, development was monitored for 100 days at 12 °C, but no parasitoid emergence occurred, indicating that this temperature is below the threshold required for *Te. remus* development under constant exposure.

**Figure 1:**
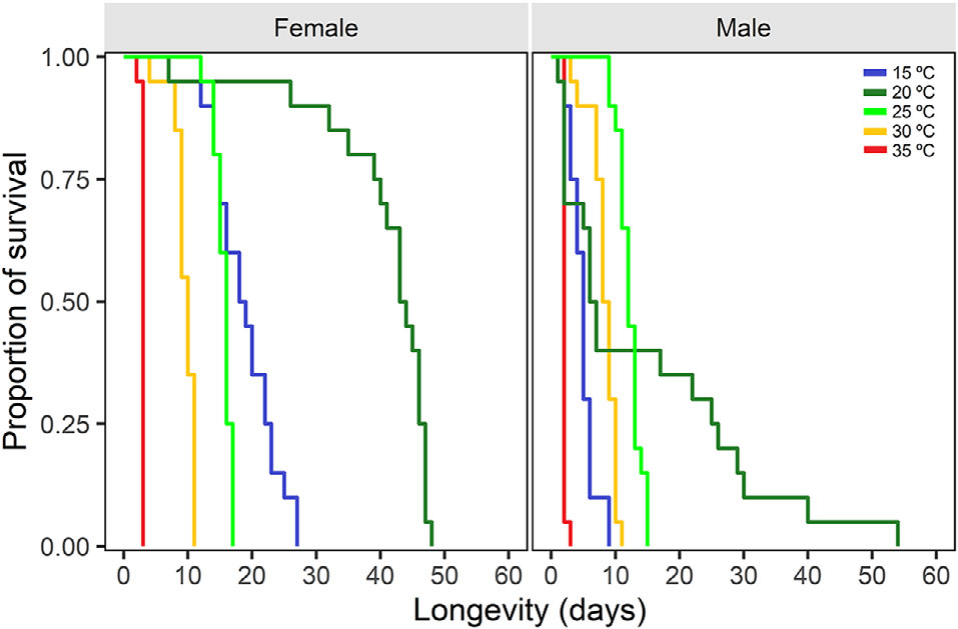
Longevity of *Telenomus remus* adults reared at different constant temperatures regimes. Survival curves were generated with the non‐parametric analysis of Kaplan–Meier.

The sex ratio of *Te. remus* was significantly influenced by temperature (χ^2^ = 18.09; df = 4; *P* < 0.01). At low temperatures (15 and 20 °C), a higher proportion of females emerged compared to males, with the high ratio observed at 15 °C (0.94 ± 0.03) (Table 2). Longevity was significantly influenced by temperature (χ^2^ = 491.45; df = 4; *P* < 0.001), sex (χ^2^ = 149.39; df = 1; *P* < 0.001), and the interaction between these factors (χ^2^ = 53.49; df = 4; *P* < 0.001). Females exhibited greater longevity than males at all evaluated temperatures (Fig. 1). The longest lifespan for both sexes occurred at 20 °C, with females living an average of 40.60 ± 2.17 days and males 14.55 ± 4.07 days. Conversely, at 35 °C, females and males survived only 2.95 ± 0.05 and 2.05 ± 0.11 days, respectively. Survival analysis confirmed these findings, showing significant differences in longevity for females (χ^2^ = 178; df = 4; *P* < 0.001) and males (χ^2^ = 94.5; df = 4; *P* < 0.001) across the temperature range tested (Fig. 1).

### 3.2 Selection and performance of mathematical models

**Figure 2:**
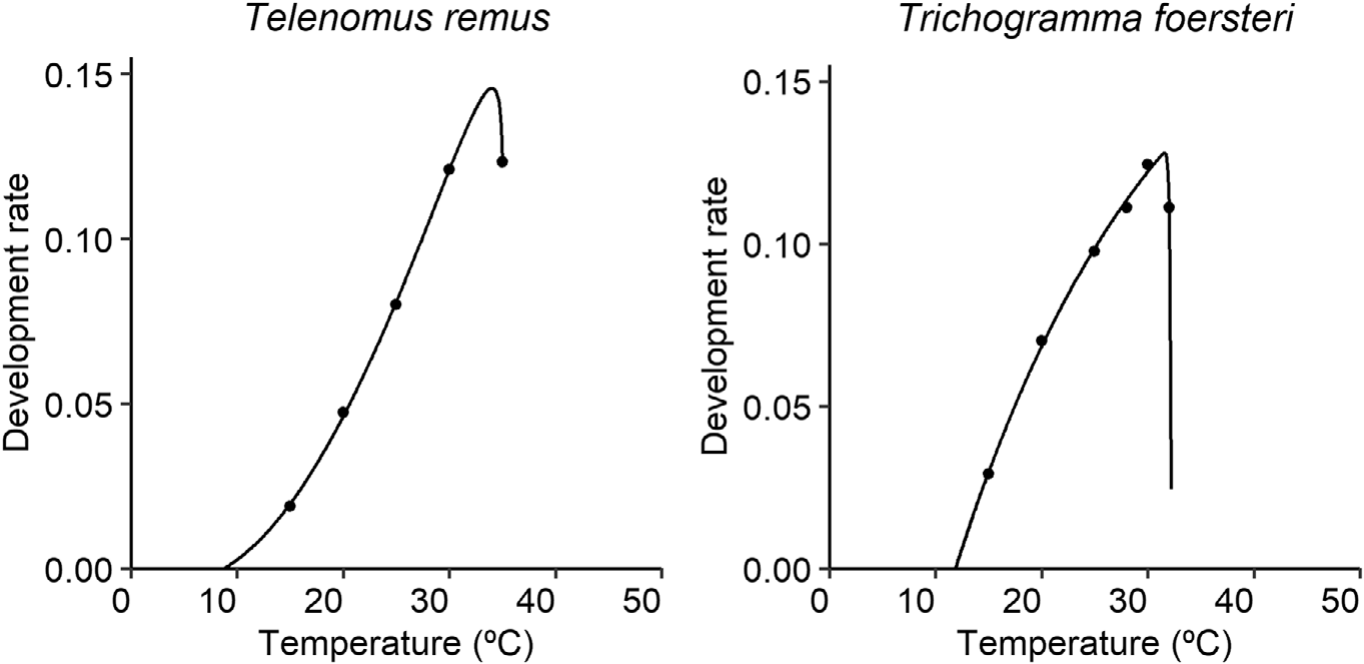
Fitting of the mathematical models selected to describe the temperature‐dependent development rate of *Telenomus remus* and *Trichogramma foersteri* for egg‐adult life cycle.

The fit and performance of the 12 evaluated models evaluated varied according to the development data of each species (see Figs S1 and S2). Based on S, ΔAIC, and ΔBIC values, the models with the best fit for *Te. remus* and *T. foersteri* were Brière‐2 and Shi, respectively (Table 3) (Fig. 2). The estimated thermal thresholds differed depending on the mathematical model used. For example, for *Te. remus*, T_L_ ranged from −17.89 to 12.66 °C, while T_H_ ranged from 35.04 to 46.79 °C. For *T. foersteri*, T_L_ varied between 5.88 and 11.87 °C, and T_H_ between 32.22 and 41.55 °C. The linear model estimated T_L_ as 11.16 for *Te. remus* and 9.55 °C for *T. foersteri*, with a thermal constant of 176.94°‐days for *Te. remus* and 162.43 for *T. foersteri* to complete the egg‐to‐adult development (Table 4).

**Table 3:** Performance of the 12 mathematical models used to describe the temperature‐dependent development rate of *Telenomus remus* and *Trichogramma foersteri* based on the standard error of the regression (*S*), the Akaike Information Criterion (AIC), and Bayesian Information Criterion (BIC)

Model	Egg to adult					
	*Telenomus remus*			*Trichogramma foersteri*		
	*S* (10^−3^)	ΔAIC^†^	ΔBIC	*S* (10^−3^)	ΔAIC^†^	ΔBIC
Linear	10.94	20.25	21.03	4.80	—^‡^	—^‡^
Beta‐16	3.22	7.99	8.38	5.80	7.49	7.91
β type	3.34	7.20	7.20	6.97	9.28	9.49
Brière‐1	5.70	13.70	14.09	5.30	6.42	6.84
Brière‐2	**1.67**	**0.00**	**0.00**	6.30	8.06	8.27
Kontodimas	6.25	14.63	15.02	5.71	7.32	7.74
Lactin‐1	3.70	9.39	9.78	6.98	9.72	10.14
Lactin‐2	3.49	7.33	7.33	5.71	6.88	7.09
Logan‐6	5.24	11.40	11.40	8.55	11.73	11.94
Performance‐2	6.35	13.32	13.32	5.45	6.32	6.53
Shi	6.35	15.52	15.13	**3.85**	**0.00**	**0.00**
Taylor	4.05	10.30	10.69	6.23	8.36	8.78

^†^ Bold *S*, ΔAIC and ΔBIC values indicate models with better performance (ΔAIC ≤2).

^‡^ Values not calculated because of the differences in the number of samples due to the exclusion of the data obtained at 35 °C when fitting the linear model.

**Table 4:** Parameter values (± SE) for each mathematical model used to describe the relationship between temperature and development rate of *Telenomus remus* and *Trichogramma foersteri*

Model^†^	Egg‐adult life cycle	
	*Telenomus remus*	*Trichogramma foersteri*
Linear		
*a*	−0.06 ± 0.01	−0.05 ± 0.01
*b* (10^−3^)	5.65 ± 0.69	6.15 ± 0.39
*T*_*L*_	11.16	9.55
*K*	176.94	162.43
Beta‐16		
*c*_*m*_ (10^−1^)	1.30 ± 0.04	1.17 ± 0.04
*T*_*L*_	−17.89 ± 34.72	5.88 ± 12.11
*T*_*H*_	40.07 ± 1.61	40.62 ± 6.11
*T*_*opt*_	32.96 ± 0.35	30.06 ± 1.39
β type		
*ρ* (10^−2^)	0.14 ± 0.02	0.34 ± 0.07
*a*	4.16 ± 0.09	3.89 ± 0.18
*β*	3.90 ± 0.22	3.30 ± 0.35
*T*_*H*_	41.60	38.90
*T*_*opt*_	34.05	29.90
Brière‐1		
*a* (10^−5^)	6.51 ± 1.00	7.65 ± 1.13
*T*_*L*_	11.73 ± 1.32	9.20 ± 1.52
*T*_*H*_	40.53 ± 1.46	36.11 ± 0.99
*T*_*opt*_	33.80	30.00
Brière‐2		
*a* (10^−4^)	1.69 ± 0.09	1.20 ± 0.56
*m*	14.22 ± 10.32	3.98 ± 3.14
*T*_*L*_	8.72 ± 1.14	7.5 ± 4.26
*T*_*H*_	35.04 ± 11.33	33.81 ± 2.82
*T*_*opt*_	34.00	30.00
Kontodimas		
*α* (10^−5^)	1.63 ± 0.54	1.99 ± 0.59
*T*_*L*_	9.50 ± 1.45	7.47 ± 1.37
*T*_*opt*_	34.40	30.20
*T*_*H*_	46.79 ± 3.04	41.55 ± 2.15
Lactin‐1		
*ρ*	0.17 ± 0.008	0.16 ± 0.01
Δ	5.60 ± 0.28	6.07 ± 0.60
*T*_*H*_	38.53 ± 0.45	35.94 ± 0.99
*T*_*opt*_	32.90	29.90
Lactin‐2		
*ρ*	0.16 ± 0.02	0.59 ± 0.07 (10^−2^)
Δ	6.11 ± 0.90	1.88 ± 1.45
*𝜆*	−0.008 ± 0.01	−1.06 ± 0.01
*T*_*L*_	5.70	10.10
*T*_*H*_	39.03 ± 1.02	38.66 ± 4.70
*T*_*opt*_	32.90	30.10
Logan‐6		
*ρ*	0.17 ± 1.70	0.16 ± 2.78
Δ	5.46 ± 56.04	5.91 ± 106.25
*φ* (10^−1^)	0.08 ± 29.07	0.19 ± 1101.93
*T*_*H*_	38.53 ± 1.83	35.94 ± 2.89
*T*_*opt*_	32.90	29.90
Performance‐2		
*cc* (10^−3^)	6.78 ± 0.89	6.56 ± 0.76
*K*	3.43 ± 3.14	0.63 ± 0.54
*T*_*L*_	12.63 ± 1.11	10.10 ± 1.24
*T*_*H*_	35.49 ± 4.47	34.36 ± 1.81
*T*_*opt*_	34.20	30.2
Shi		
*c*	9.22 ± 1.26	0.20 ± 0.08
*K*_1_ (10^−2^)	0.07 ± 7.53	4.97 ± 3.14
*K*_2_	2.95 ± 0.04	8.57 ± 5.33
*T*_*L*_	12.66 ± 11.31	11.87 ± 1.00
*T*_*H*_	35.57 ± 0.09	32.22 ± 1.39
*T*_*opt*_	34.20	31.60
Taylor		
*Rm* (10^−1^)	1.26 ± 0.03	1.16 ± 0.03
*T*_*L*_	9.39 ± 0.66	9.28 ± 1.07
*T*_*opt*_	33.44 ± 0.79	29.99 ± 1.17

^†^ *T*_*L*_. *T*_*H*_, *T*_*opt*_ are, respectively, the lower temperature threshold, upper temperature threshold and the optimum temperature for development (°C), *K* is the thermal constant. The remaining parameters are fitted coefficients.

Some models produced unrealistic thermal thresholds. For *Te. remus*, the Beta‐16 model estimated a T_L_ of −17.89 °C, and the Kontodimas model estimated a T_H_ of 46.79 °C. Despite these discrepancies, certain models provided acceptable thresholds, consistent with the experimental conditions and the region where the parasitoids were collected, such as the linear and Performance‐2 models for both species. The number and accuracy of the estimated thermal thresholds, along with the quality of fit (Table 5), confirmed that the Brière‐2 and Shi models were the most suitable for *Te. remus* and *T. foersteri*, respectively. Therefore, these models were employed to estimate the voltinism of *Te. remus* and *T. foersteri*.

**Table 5:** Thermal thresholds estimated by each model used to describe the temperature‐dependent development rate of *Telenomus remus* and *Trichogramma foersteri*, and their accuracy inferred based on the observed development times and on the known species distribution range. *T*_*L*_ and *T*_*H*_ are, respectively, the lower and upper thermal thresholds, and *T*_*opt*_ is the optimum temperature

Models	Number of estimated thermal thresholds	Accuracy^†^					
		*Telenomus remus*			*Trichogramma foersteri*		
		*T*_*L*_	*T*_*opt*_	*T*_*H*_	*T*_*L*_	*T*_*opt*_	*T*_*H*_
Linear	1	+	•	•	+	•	•
Beta‐16	3	−	+	−	+	+	−
β type	2	•	+	−	•	+	−
Brière‐1	3	+	+	−	+	+	−
Brière‐2	**3**	+	+	+	+	+	+
Kontodimas	3	+	+	−	+	+	−
Lactin‐1	2	•	+	−	•	+	−
Lactin‐2	3	−	+	−	+	+	−
Logan‐6	2	•	+	−	•	+	−
Performance‐2	3	+	+	+	+	+	+
Shi	**3**	+	+	+	+	+	+
Taylor	2	+	+	•	+	+	•

^†^ +, yes; −, no; •, parameter not estimated by the model.

### 3.3 Voltinism under current and projected climate conditions

**Figure 3:**
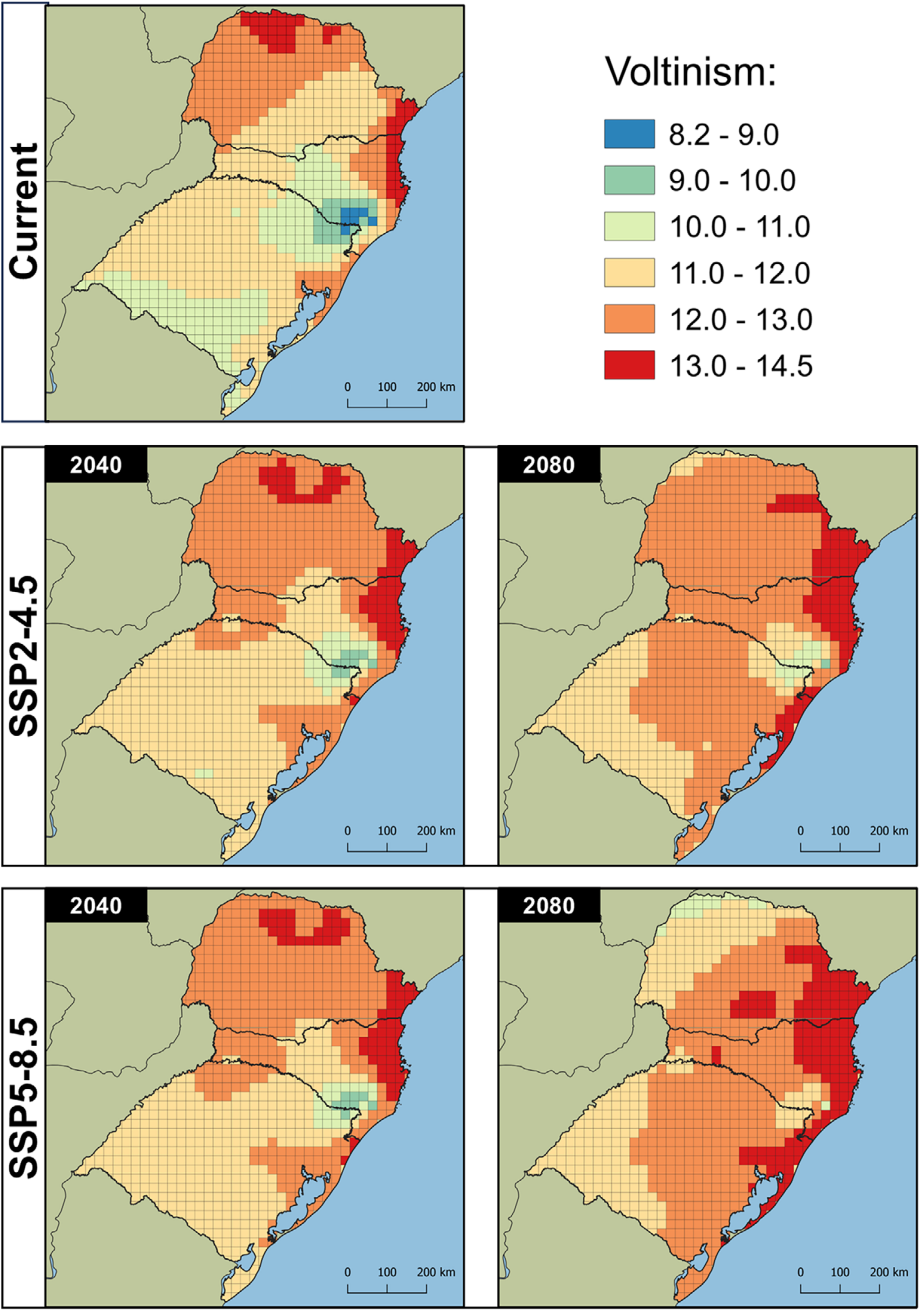
Voltinism of *Telenomus remus* in southern Brazil estimated by the model Brière‐2. Future climate projections for 2040 and 2080 derived from the climate change scenarios SSP‐4.5 and SSP‐8.5.

**Figure 4:**
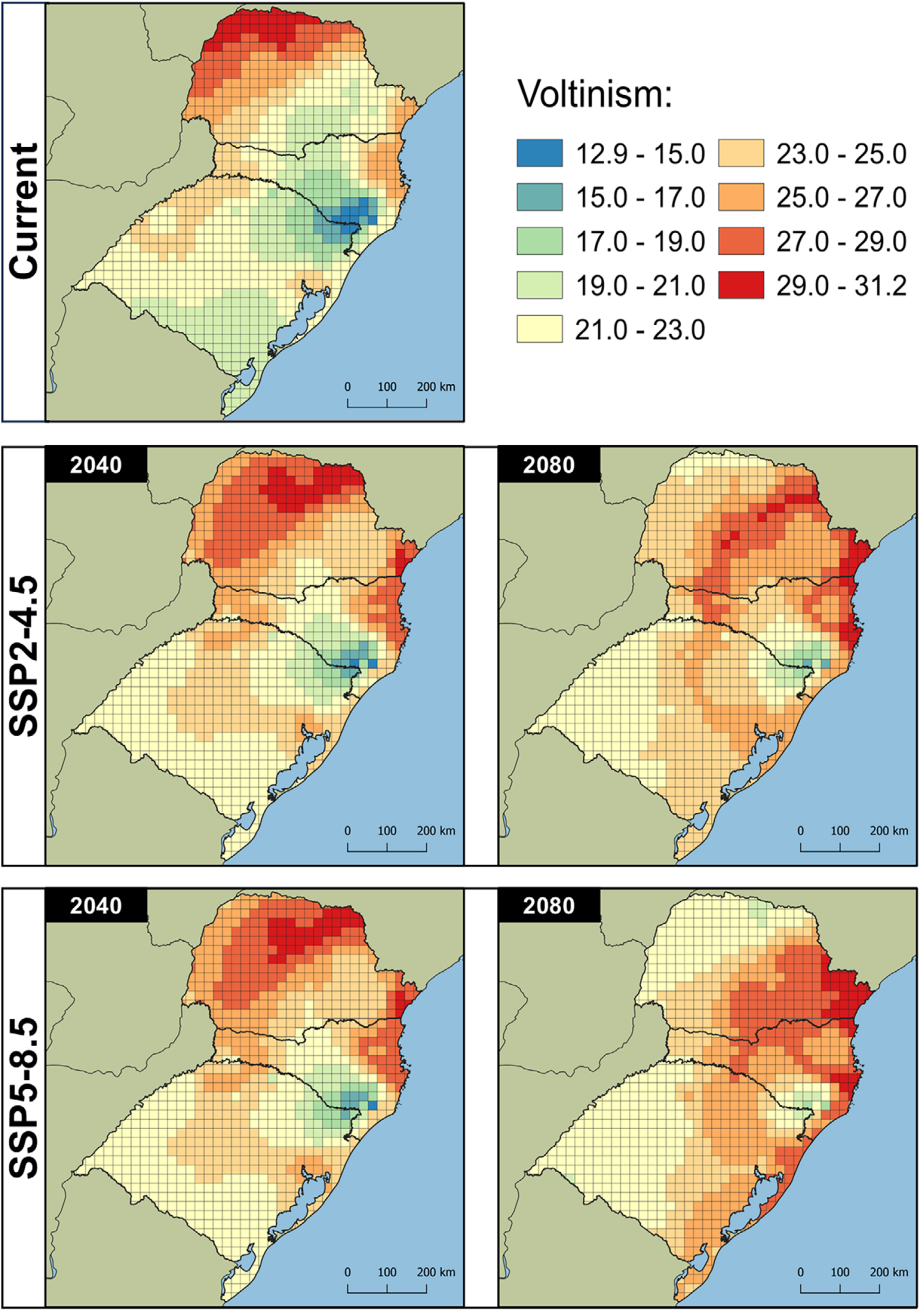
Voltinism of *Trichogramma foersteri* in southern Brazil estimated by the model Shi. Future climate projections for 2040 and 2080 derived from the climate change scenarios SSP‐4.5 and SSP‐8.5.

The lower and upper thermal thresholds estimated by the Brière‐2 model for *Te. remus* were 8.72 °C and 35.04 °C, respectively. Using the Shi equation, the T_L_ estimated for *T. foersteri* was 11.87 °C, while the T_H_ was 32.22 °C. Under current climatic conditions, the number of generations in the study area varied from 8.27 to 13.89 for *Te. remus* and from 12.94 to 30.87 for *T. foersteri* (Figs 3 and 4). Both species exhibited a similar geographic pattern in voltinism, with fewer generations in high‐altitude areas of southern Santa Catarina and northern Rio Grande do Sul, and more generations in warmer regions, such as western, northwestern, and coastal Paraná, as well as northeastern Santa Catarina.

When the parasitoids' voltinism was estimated under different climate change scenarios and periods, an increase in the number of generations was observed for both species. In the SSP2‐4.5/2040 scenario, the number of generations ranged from 9.11 to 14.32 for *Te. remus* and from 14.67 to 30.38 for *T. foersteri*. By 2080, under the same scenario, the estimated number of generations increased to 9.98–14.52 for *Te. remus* and 16.52–31.20 for *T. foersteri*. Under the SSP5‐8.5/2040 scenario, the range was 9.23–14.37 for *Te. remus* and 14.92–30.41 for *T. foersteri*. Finally, in the SSP5‐8.5/2080 scenario, the number of generations rose to 10.53–14.47 for *Te. remus* and 18.61–30.89 for *T. foersteri*.

The SSP2‐4.5/2080 scenario showed the greatest increase in the number of generations. Notably, the higher voltinism observed under SSP2‐4.5 for both species suggests that the temperature rise projected in the SSP5‐8.5 scenario (2.4 °C for 2041–2060 and 4.4 °C for 2081–2100) may exceed the upper thermal thresholds tolerated by the parasitoids, thereby reducing voltinism in warmer regions (Figs 3 and 4). Conversely, in colder regions of the study area, this temperature increase may promote the development of both species, leading to a higher number of annual generations.

**Figure 5:**
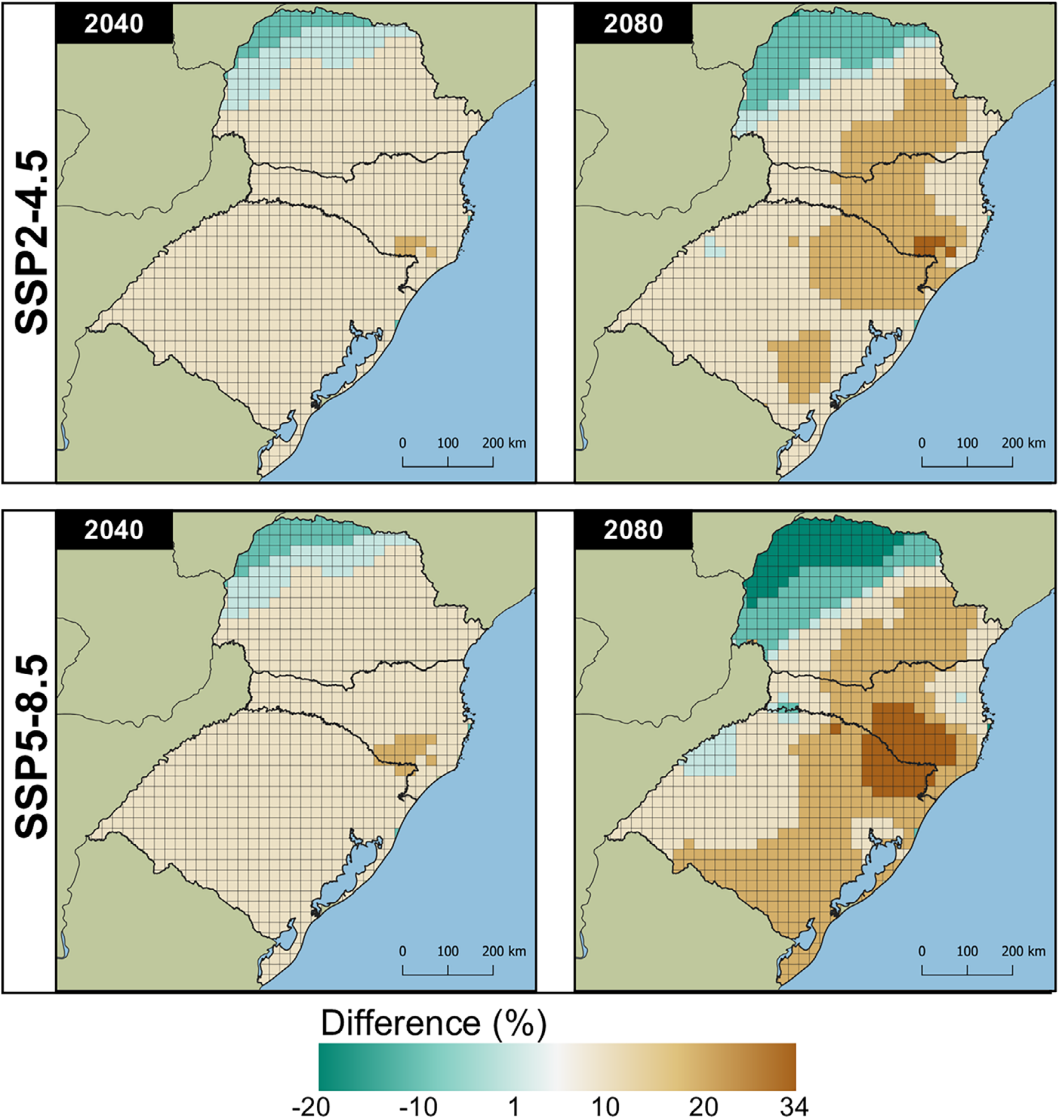
Differences (%) between current and future projections of voltinism for *Telenomus remus* in southern Brazil estimated with the model Brière‐2. Future climate projections for 2040 and 2080 derived from the climate change scenarios SSP‐4.5 and SSP‐8.5. Negative and positive values refer to decrease and increase in the number of generations, respectively.

**Figure 6:**
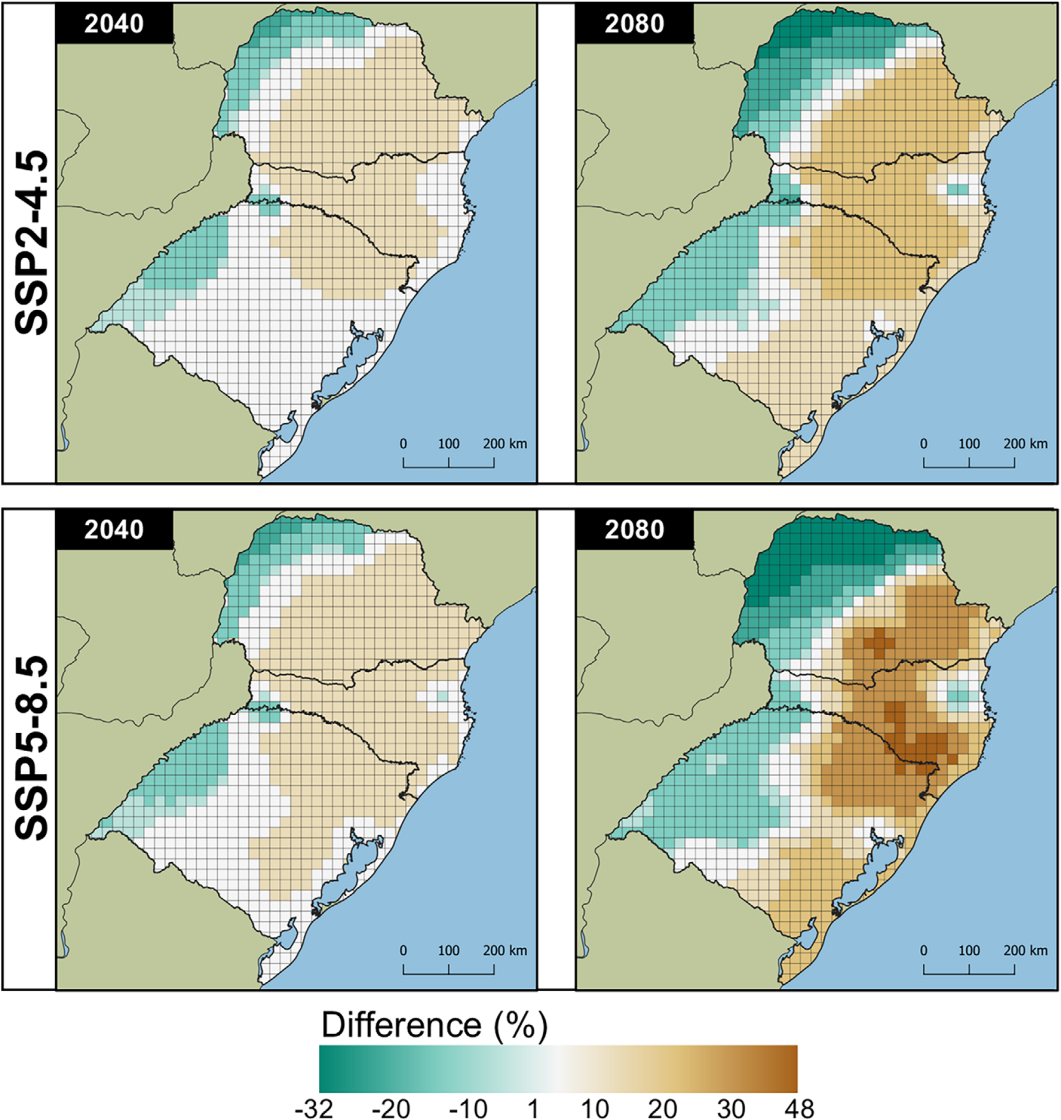
Differences (%) between current and future projections of voltinism for *Trichogramma foersteri* in southern Brazil estimated with the model Shi. Future climate projections for 2040 and 2080 derived from the climate change scenarios SSP‐4.5 and SSP‐8.5. Negative and positive values refer to decrease and increase in the number of generations, respectively.

Regardless of the species evaluated, the models projected an increase in the number of generations in colder regions under future climate scenarios. The SSP5‐8.5/2080 scenario showed the highest increases, with up to 33.98% (2.81 generations) for *Te. remus* and 47.07% (6.22 generations) for *T. foersteri*. In the same scenario, the largest decrease in the number of generations was observed in warmer regions, with potential reductions of up to 19.01% (−2.51 generations) for *Te. remus* and 31.22% (−9.59 generations) for *T. foersteri* (Figs 5 and 6).

### 3.4 Number of days exceeding the thermal thresholds

**Figure 7:**
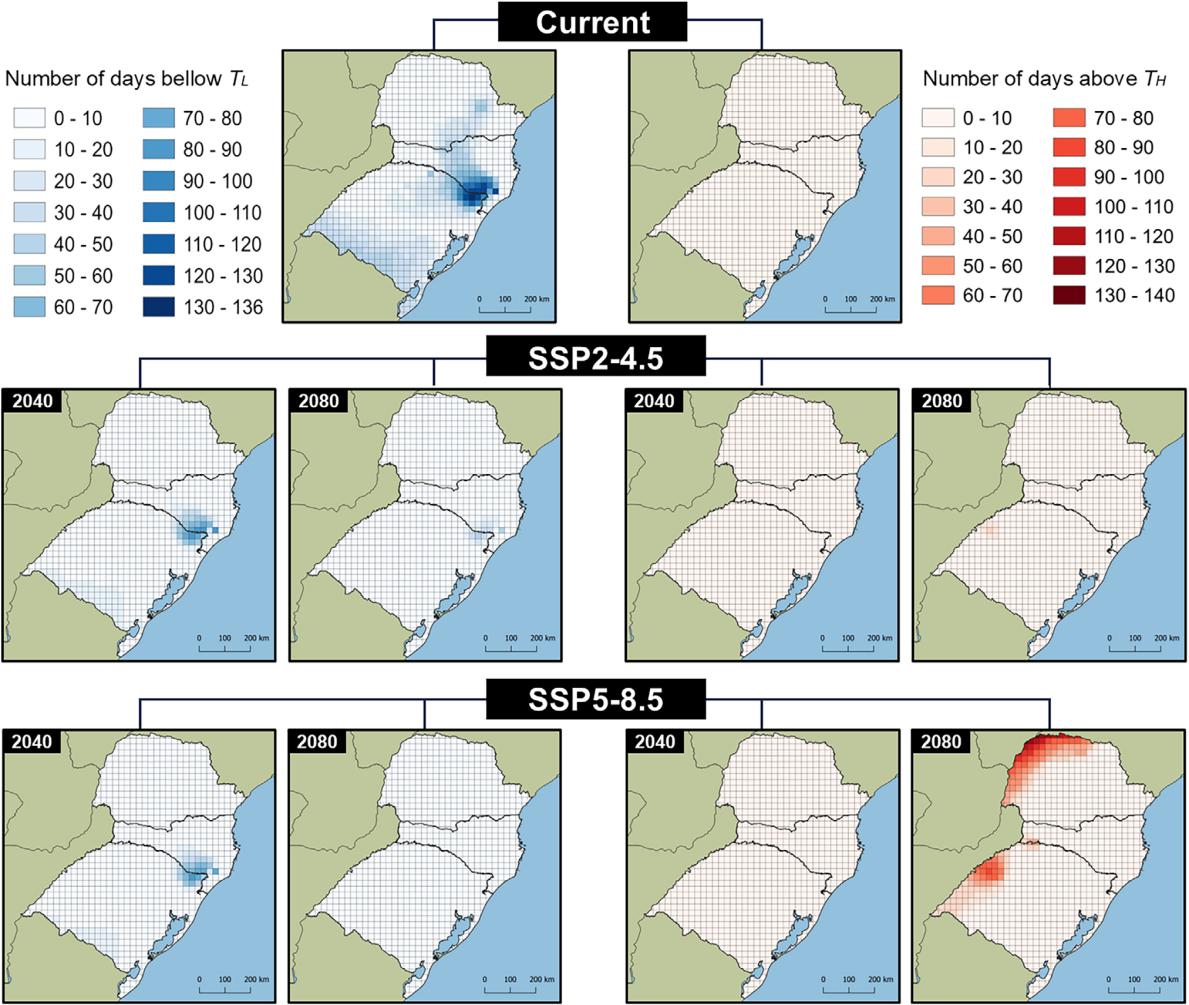
Number of days in the year in which the daily temperature was below the lower thermal threshold of 8.72 °C, and above the upper thermal threshold of 35.04 °C estimated for *Telenomus remus* in southern Brazil.

Under current climatic conditions, the daily minimum temperature falls below the estimated T_L_ for *Te. remus* for up to 136 days per year. This is projected to decrease to as few as 10 days in future climate scenarios, such as SSP5‐8.5/2080 (Fig. 7). The greatest variations in relation to T_L_ were observed in the colder regions of southern Brazil. Currently, no temperatures equal to or exceeding the estimated T_H_ for *Te. remus* (35.04 °C) have been recorded. However, under future climate change scenarios, up to 140 annual days are expected to reach or exceed this threshold (Fig. 7), particularly in the northwestern regions of Paraná and Rio Grande do Sul.

**Figure 8:**
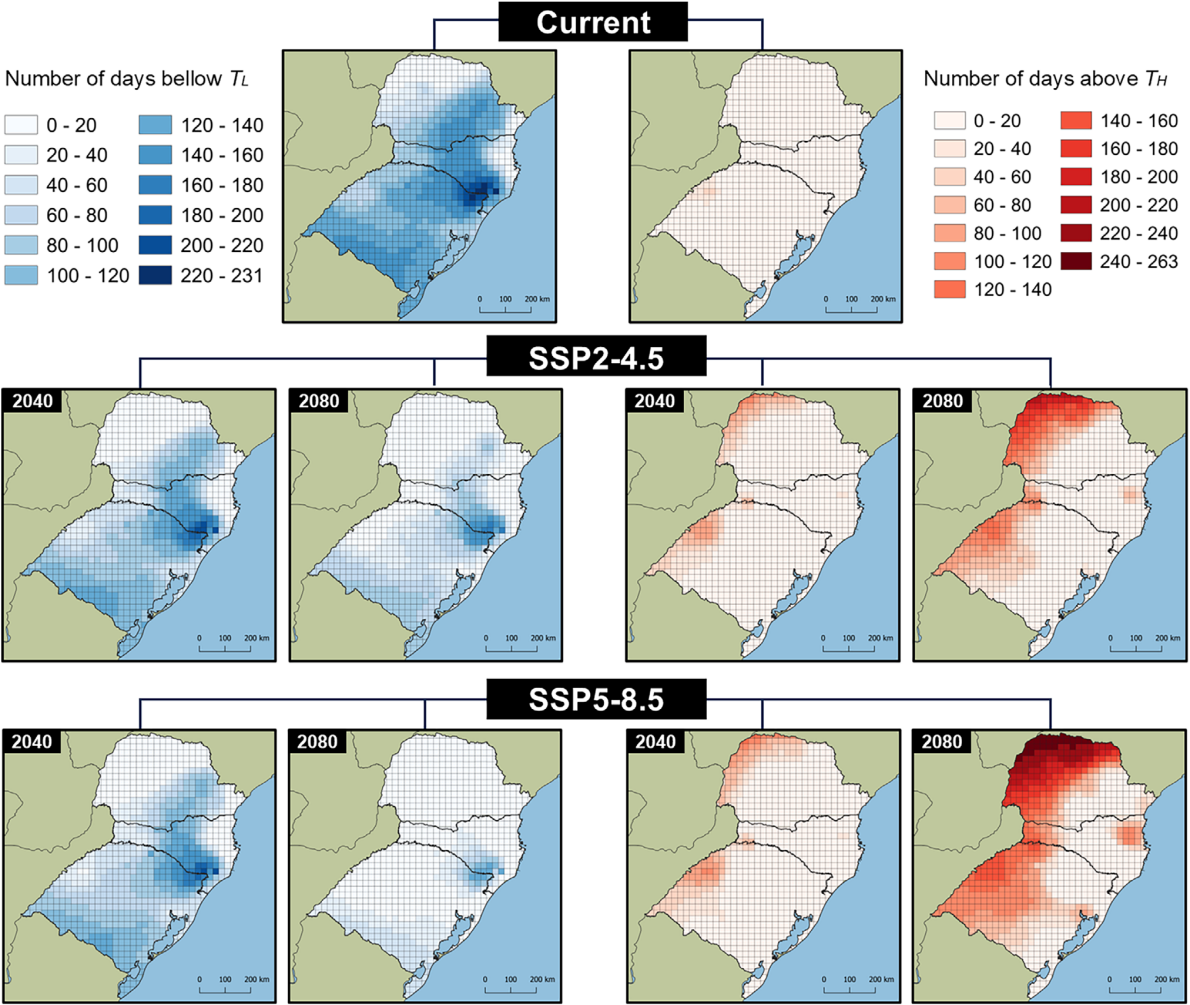
Number of days in the year in which the daily temperature was below the lower thermal threshold of 11.87 °C, and above the upper thermal threshold of 32.22 °C estimated for *Trichogramma foersteri* in southern Brazil.

For *T. foersteri*, the T_L_ is reached for up to 231 days per year under current climatic conditions (Fig. 8). Even in future projections, the number of days remains high, with the highest value recorded in the SSP5‐4.5/2040 scenario (207 days). Currently, the T_H_ for *T. foersteri* is reached on 50 days per year, and projections suggest this could increase to as many as 263 days in warmer areas under future climatic conditions.

## 4 DISCUSSION

Numerous studies have shown that temperature significantly influences insect development and survival. However, because the response to temperature varies across species, research on its effects on insect biology remains essential.^21^ This study examined the direct impact of temperature on the development of a lineage of *Te. remus* adapted to the climatic conditions of southern Brazil. Additionally, phenological models were fitted to explore the relationship between temperature and development in *Te. remus* and *T. foersteri*. Finally, potential climate change impacts on the voltinism of these parasitoids were assessed.

Temperature influenced the developmental time and survival of *Te. remus*, as evidenced by the parasitoid's response to extreme temperatures. A notable difference of 44.6 days in the average development time was observed between individuals reared at 15 and 35 °C. Lower temperatures reduced parasitism, while higher temperatures (*e.g*., 35 °C) stimulated parasitic activity. However, at these temperature extremes, the lowest emergence rates were recorded, particularly at 15 °C. These findings indicate that the population growth of *Te. remus* may be constrained in regions where temperatures frequently approach or fall below 15 °C. Cold conditions can cause metabolic damage, such as osmotic stress, and reduce the degree days accumulated for complete development, leading to slower growth rates.^24^ Additionally, low temperatures may prematurely interrupt feeding,^52^ increasing larval mortality and reducing emergence rates, as observed in this study. Conversely, *Te. remus* appears to thrive at higher temperatures (up to 35 °C), particularly regarding development time and parasitism. At elevated temperatures, developmental changes likely result from metabolic adjustments in the insect.^45^, ^53^, ^54^ Notably, the development time decreases as temperature rises, up to a threshold beyond which survival becomes compromised.^55^

Although an emergence rate of 78.16% was observed in this study at 35 °C, previous research reported no emergence at this temperature^55^ or only 5.83% at 34 °C.^56^ Additionally, a higher proportion of females was observed at 15 °C, whereas another study under similar conditions reported a predominance of males.^55^ When a different field‐collected lineage of *Te. remus* from Piracicaba, SP, Brazil, was analyzed, considerably lower parasitism rates were recorded compared to this study.^57^ Despite the close timing of collection for both lineages, the regions are approximately 500 km apart^4^ and experience significantly different climatic conditions. Although the lineages likely share a common origin,^4^ distinct performance among them may result from adaptation to local environmental conditions,^26^ which could explain the differences observed in our study.

The most effective approach for conducting studies with phenological models to explain insect development involves testing multiple models and using a multi‐criteria process to select the most suitable equation.^18^, ^20^, ^21^ In addition to evaluating the quality of the model fit, we also considered the precision of the estimated thermal thresholds. Among the 12 models tested, Brière‐2 and Shi were the ones that best described the relationship between temperature and developmental rate for *Te. remus* and *T. foersteri*, respectively. Although the same equations were fitted for both parasitoids, each species responded differently to the functions, leading to the selection of distinct models. This variation among models highlights that model selection and evaluation should be carried out on a case‐by‐case basis.^21^ Few models met the pre‐established criteria, indicating that fitting and selecting the most appropriate model can be particularly challenging when working with egg parasitoids. These difficulties may help explain why, despite the importance of such studies, relatively few works have focused on model selection for egg parasitoid Hymenoptera. This emphasizes the need for a thorough evaluation of equations for this insect group.

Overall, our findings indicate that the selected models provided parameter estimates consistent with empirical observations. For *Te. remus*, the upper thermal threshold and optimal temperature estimated by the Brière‐2 model aligned well with laboratory data. A similar pattern was observed for *T. foersteri* using the Shi model. However, some discrepancies were noted in the estimation of the lower thermal threshold for *Te. remus*. Although no parasitoid emergence was recorded under laboratory conditions at a constant temperature of 12 °C over 100 days, the Brière‐2 model estimated a lower threshold of 8.72 °C for this species. For *T. foersteri*, the Shi model estimated a lower threshold of 11.87 °C, consistent with another study in which no development of *T. foersteri* was observed at 10 °C when reared on olive moth eggs, *Palpita forficifera* (Munroe).^11^ Despite these discrepancies, the Brière‐2 model appears to provide a more realistic estimate of the lower threshold. The thermal thresholds estimated by the model for *Te. remus* (8.72 °C) and *T. foersteri* (7.5 °C) represent temperatures commonly experienced by the parasitoids in the field. This supports the notion that, despite our findings and those of other studies, *Te. remus* and *T. foersteri* are capable of surviving at 12 °C and 10 °C, respectively, under natural field conditions. Moreover, they likely withstand even lower temperatures, as the collection region frequently experiences low winter temperatures,^15^ with frosts being common during this season.^16^

The estimated lower thresholds may help explain the difficulty in collecting larger numbers of both parasitoid species in the field, despite repeated sampling efforts. When temporarily exposed to temperatures below their lower thermal threshold, development is halted but resumes once temperatures rise above the lower threshold.^24^ However, even if the parasitoids are capable of tolerating lower temperatures, such as those experienced in southern Brazil, prolonged exposure may negatively impact their development, survival, and reproduction.^24^ Therefore, although parasitoids can endure such conditions, severe winters may disrupt their populations, leading to a significant decline in abundance in the region.

Non‐linear models are widely regarded as more appropriate for generating reliable estimates in studies assessing the impacts of climate change on insect development,^18^, ^20^, ^23^ because they incorporate an upper thermal threshold into their function. Upper thermal thresholds in insects tend to show less variation than lower thresholds,^25^ as they are confined to a narrow range that usually follows the optimal temperature. In our study, we observed a 3 °C difference between the upper thermal thresholds estimated for the two parasitoid species, consistent with laboratory data. This difference proved sufficient to illustrate how identical global warming scenarios could have distinct impacts on each species. For example, *T. foersteri* may experience developmental constraints in certain regions of the study area, as higher temperatures could surpass its upper threshold (32.22 °C) for up to 263 days per year.

In colder regions, rising temperatures could increase the number of generations of both parasitoids, as their estimated lower thresholds would be exceeded more frequently. Currently, these areas, which include mountainous regions in southern Santa Catarina, northern Rio Grande do Sul, and southern Paraná, are subject to frequent frosts and occasional snowfalls during winter,^16^ resulting in fewer generations for *Te. remus* and *T. foersteri* under present climatic conditions. Consequently, the region's climatic heterogeneity contributed to the variability in the predicted number of generations for both parasitoids across different climate scenarios. In several studies, geographical location consistently emerges as the primary factor explaining variation in the predicted voltinism of insects.^22^, ^23^, ^58^, ^59^

Host‐parasitoid interactions play a key role in regulating arthropod populations and maintaining ecosystem balance. However, climate change, particularly global warming, poses significant challenges to these interactions. While our study focused on the thermal limits of parasitoids, it is likely that hosts, such as *S. frugiperda*, exhibit distinct thermal responses. Parasitoids often have lower thermal requirements than their hosts, making them more vulnerable to rising global temperatures.^25^ Moreover, extreme temperatures can disrupt host‐parasitoid synchronization, potentially leading to mismatches that alter ecological dynamics.^24^ Such mismatches may shorten the window of host susceptibility, causing premature emergence of either the host or parasitoid, which in turn affects their fitness and population dynamics. Future studies integrating the thermal responses of both hosts and parasitoids will provide a more comprehensive understanding of these interactions and improve predictions of climate change impacts on biological control success.

The thermal ecology of most economically important species in agriculture, horticulture, and forestry remains largely unexplored.^54^ This study is the first to assess the potential impact of climate change on the development of *Te. remus* and *T. foersteri* in Brazil. Beyond temperature, other environmental factors can significantly influence the number of generations of these species, highlighting the importance of validating models under natural conditions as a critical step in the modeling process. Nevertheless, we recognize the challenges of obtaining such field data, which likely contributes to the limited number of studies addressing the effects of fluctuating temperatures on parasitoids.^25^

Our data demonstrated that temperature plays a significant role in the development and survival of the *Te. remus* lineage collected in southern Brazil. Among the 12 models tested, Brière‐2 and Shi are recommended for describing the temperature‐dependent development rates of *Te. remus* and *T. foersteri*, respectively. Based on the thermal thresholds estimated by these models, both parasitoids can develop across a broad temperature range. These findings are essential for optimizing parasitoid rearing laboratory settings, improving mass production in biofactories, and planning future field releases. Climate change is expected to affect parasitoid development in southern Brazil, with impacts varying according to the region's climatic conditions. While some areas may become more conducive to parasitoid development, others could see a reduction in voltinism. This study provides valuable insights into the temperature‐driven dynamics of Hymenopteran egg parasitoids and highlights the potential consequences of climate change on these biological control agents. The methods applied, grounded in the biology of *Te. remus* and *T. foersteri*, offer critical perspectives to improve forecasting of the ecological consequences of global warming on parasitoids.

## CONFLICT OF INTEREST

The authors have no relevant financial or non‐financial interests to disclose.

## AUTHOR CONTRIBUTIONS

All authors contributed to the study conception and design. Material preparation, data collection and analysis were performed by Fábio Sampaio. The first draft of the manuscript was written by Fábio Sampaio and Cesar Augusto Marchioro. Luis Amilton Foerster contributed critically to the review of the manuscript. All authors read and approved the final manuscript.

## Supporting information


**Figure S1.** Fitting of the mathematical models used to describe the temperature‐dependent development rate of *Telenomus remus* for egg‐adult life cycle.
**Figure S2.** Fitting of the mathematical models used to describe the temperature‐dependent development rate of *Trichogramma foersteri* for egg‐adult life cycle.

## Data Availability

The datasets generated during the current study are available from the corresponding author on reasonable request.
