# Supplementary material for: Modeling parasitoid development: climate change impacts on Telenomus remus (Nixon) and Trichogramma foersteri (Takahashi) in southern Brazil
Source: Pest Manag Sci. 2025 May 8;81(9):5335–49. doi: 10.1002/ps.8888 (PMC12332101; doi:10.1002/ps.8888)
Supplement: Supplementary file 1 — Figure S1. Fitting of the mathematical models used to describe the temperature‐dependent development rate of Telenomus remus for egg‐adult life cycle. Figure S2. Fitting of the mathematical models used to describe the temperature‐dependent development rate of Trichogramma foersteri for egg‐adult life cycle. [file PS-81-5335-s001.pdf]

Modeling parasitoid development: climate change impacts on *Telenomus remus* (Nixon) and *Trichogramma foersteri* (Takahashi) in Southern Brazil

Fábio Sampaio[1*], Cesar A. Marchioro[2], Luís A. Foerster[1]

[1] Graduate Program in Plant Production - Agronomy, Department of Plant Protection, Federal University of Paraná, Curitiba, Paraná, Brazil.

[2] Graduate Program in Natural and Agricultural Ecosystems, Department of Agriculture, Biodiversity, and Forests, Federal University of Santa Catarina (UFSC), Campus of Curitibanos, Curitibanos, Santa Catarina, Brazil.

*Corresponding author: Avenida Coronel. Francisco H. Santos, 100 – Jardim das Américas, Universidade Federal do Paraná, Campus Centro Politécnico – Setor de Ciências Biológicas – Departamento de Zoologia, Curitiba, Paraná, Brazil, Zip code 81531-980, PO box 19.020. E-mail: fabiosam09@gmail.com

Supplementary figures

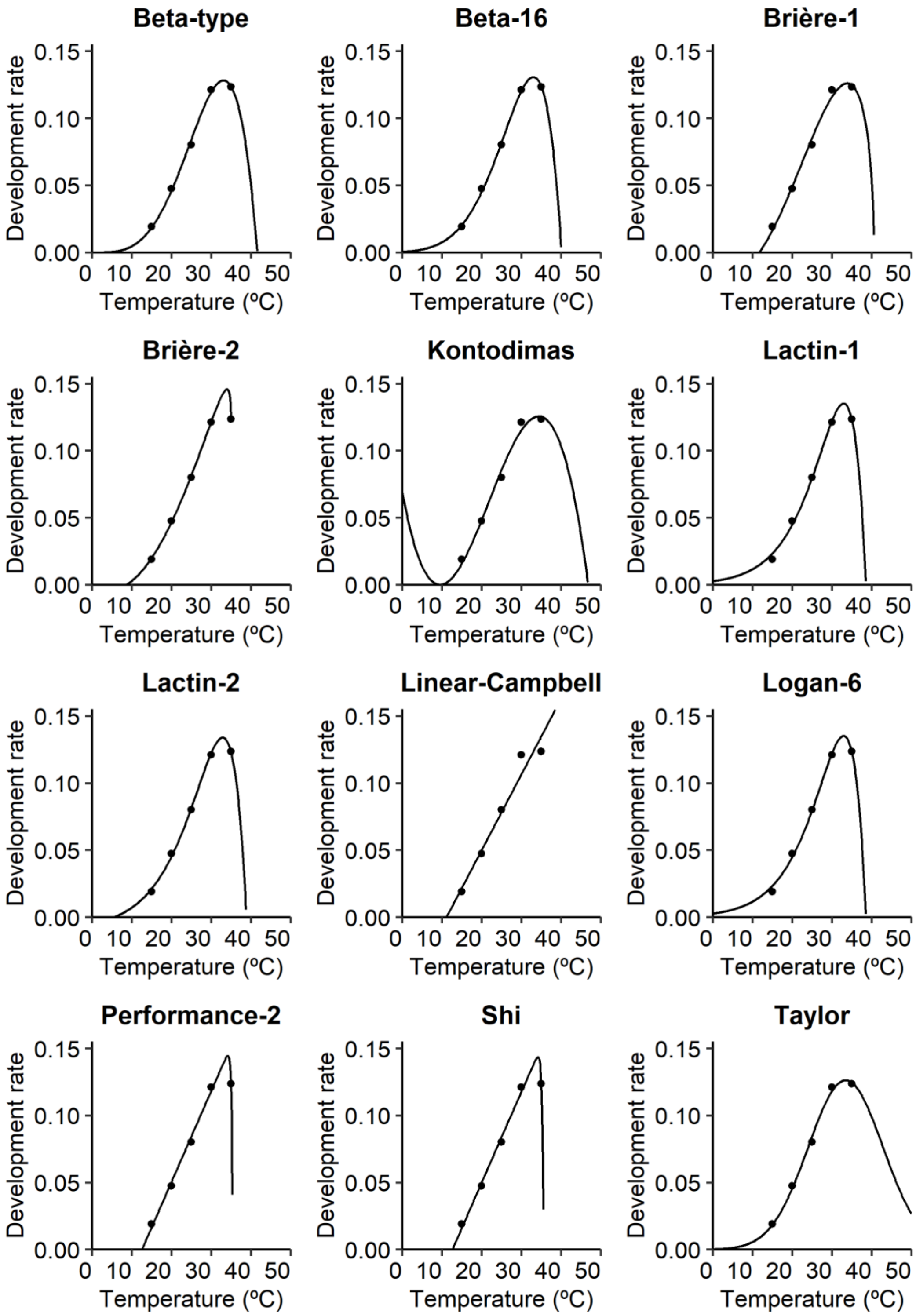

Figure S1. Fitting of the mathematical models used to describe the temperature-dependent development rate of *Telenomus remus* for egg-adult life cycle.

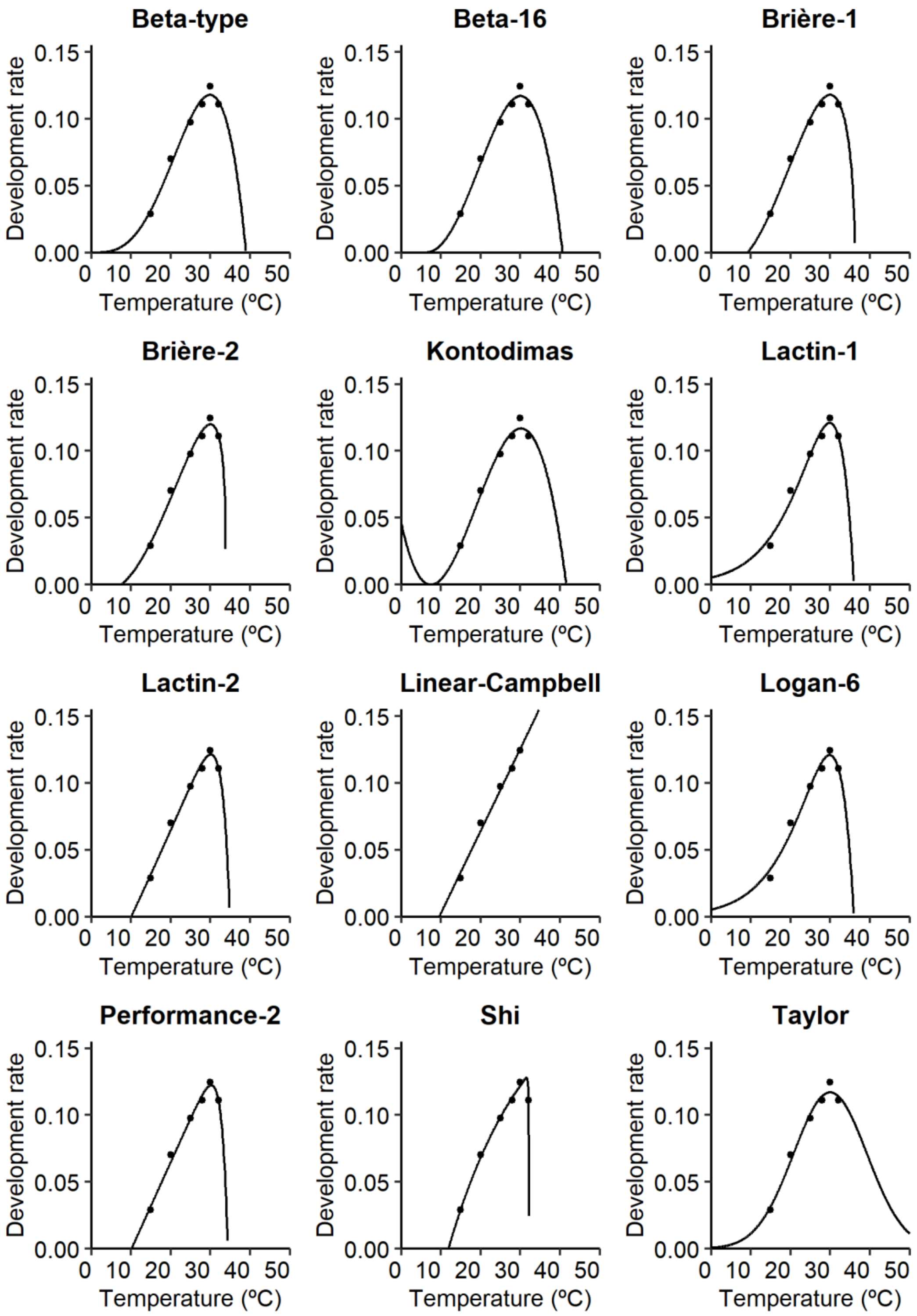

Figure S2. Fitting of the mathematical models used to describe the temperature-dependent development rate of *Trichogramma foersteri* for egg-adult life cycle.
